# Supplementary material for: TEADs, Yap, Taz, Vgll4s transcription factors control the establishment of Left-Right asymmetry in zebrafish
Source: eLife. 2019 Sep 12;8:e45241. doi: 10.7554/eLife.45241 (PMC6759317; doi:10.7554/eLife.45241)

Supplementary file 6: Position of MO (black), ASO (blue) target sequences and of mutations (red) in vgll4l, vgll4b, yap, taz, tead1a and tead3a genes (the size of introns has not been respected).

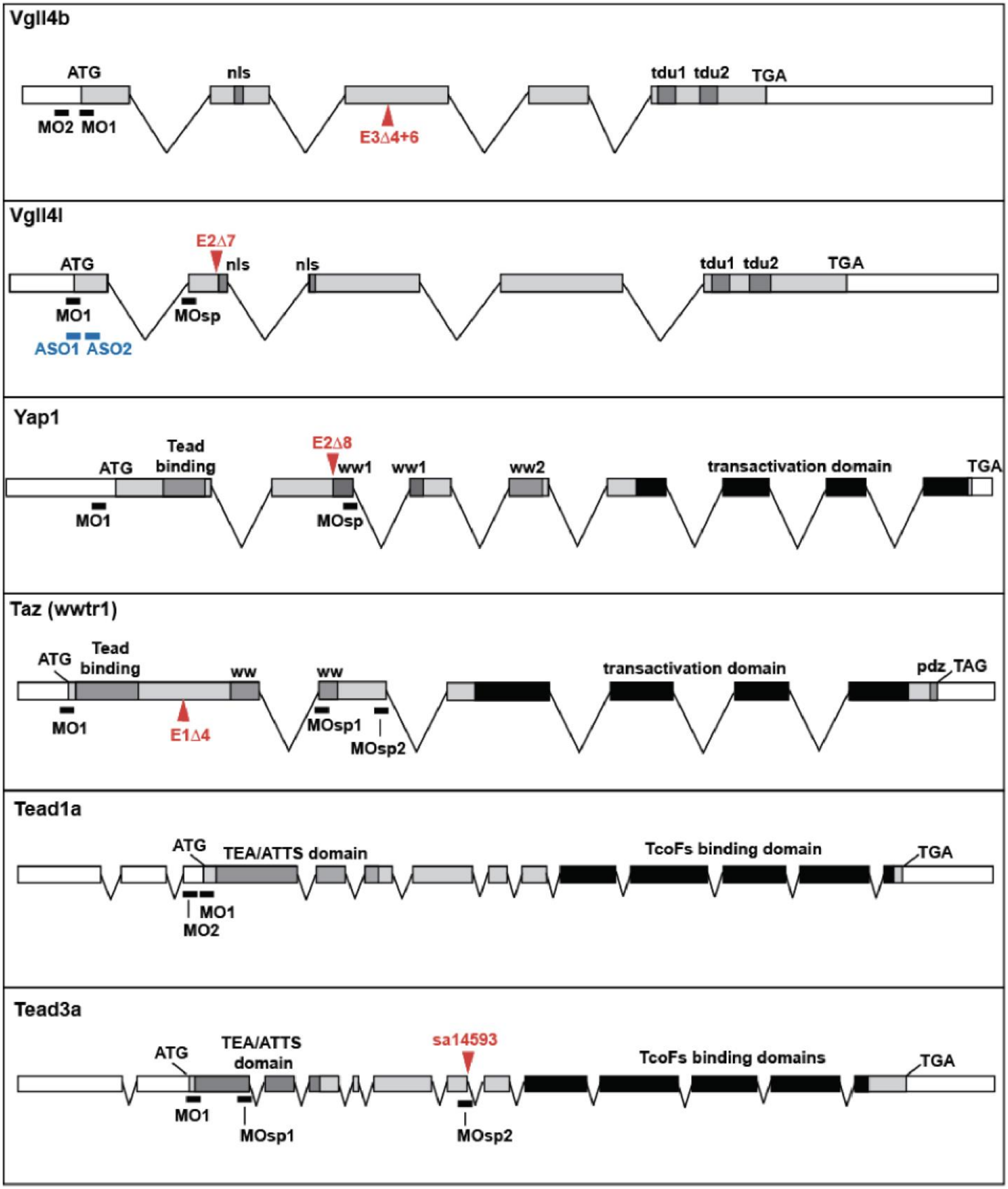

Supplement: Supplementary file 6. [file elife-45241-supp6.pdf]
