# Supplementary material for: TEADs, Yap, Taz, Vgll4s transcription factors control the establishment of Left-Right asymmetry in zebrafish
Source: eLife. 2019 Sep 12;8:e45241. doi: 10.7554/eLife.45241 (PMC6759317; doi:10.7554/eLife.45241)
Supplement: Supplementary file 7. [file elife-45241-supp7.pdf]

Supplementary File 7: Sequence of Vgll4l, Vgll4b, Yap, Taz, and Tead3a mutants.

Vgll4l E2(Δ7)

WT open reading frame is underlined. Aminoacids coded by another coding frame after the mutation site are in *red* italics.

```
WT              TGTGCGTTGAAGATTCAGGAATGGCGGTCACTAATTTCCACTACATAACCAGGATGAGCA  120
                ||||||||||||||||||||||||||||||||||||||||||||||||||||||||||||
Vgll4l E2Δ7     TGTGCGTTGAAGATTCAGGAATGGCGGTCACTAATTTCCACTACATAACCAGGATGAGCA  120
                C  A  L  K  I  Q  E  W  R  S  L  I  S  T  T  *  P  G  *  A
                 V  R  *  R  F  R  N  G  G  H  *  F  P  L  H  N  Q  D  E  Q
                  C  V  E  D  S  G  M  A  V  T  N  F  H  Y  I  T  R  M  S  S

WT              GTGGTTTCAAAGTATACATCTTAGAGGGACAGCCCAATCTGAGGAGCGAAGACAGATTCC  180
                ||||||||||||||||||||||||||||||||||||||||||||||||||||||||||||
Vgll4l E2Δ7     GTGGTTTCAAAGTATACATCTTAGAGGGACAGCCCAATCTGAGGAGCGAAGACAGATTCC  180
                V  V  S  K  Y  T  S  *  R  D  S  P  I  *  G  A  K  T  D  S
                 W  F  Q  S  I  H  L  R  G  T  A  Q  S  E  E  R  R  Q  I  P
                  G  F  K  V  Y  I  L  E  G  Q  P  N  L  R  S  E  D  R  F  R

WT              GGCACATGACCAGCGACAGAGTGCGCATGCGCCCCGCACACCCCATGAAGCGCAAGCACA  240
                |||||||       ||||||||||||||||||||||||||||||||||||||||||||||
Vgll4l E2Δ7     GGCACAT-------GACAGAGTGCGCATGCGCCCCGCACACCCCATGAAGCGCAAGCACA  233
                G  T  *         A  H  A  P  R  T  P  H  E  A  Q  A  Q  A  Q
                 A  H            R  V  R  M  R  P  A  H  P  M  K  R  K  H  S
                  H  M         T  E  C  A  C  A  P  H  T  P  *  S  A  S  T
```

peptide

```
WT              MAVTNFHYITRMSSGFKVYILEGQPNLRSEDRFRHMTSDRVRMRPAHPMKRKHSSDRGRT  60
                ||||||||||||||||||||||||||||||||||||
Vgll4l E2Δ7     MAVTNFHYITRMSSGFKVYILEGQPNLRSEDRFRHMTECACAPHTP

WT              LEERRERALSKCVANSARRSSGFSIPESPTSTWSPTASPTHLIPSPVFSSPVMDEPLALI  120

WT              KKPRPEPEKTESQNKATTQIQMRPSVITCVSSASRSTKQDCCNHSTAVSKHSYDHVEEHF  180

WT              QRSLGINYHRATSISVSVDDHFAKALGDKWLQLKASSSSCHSSSSSSSSSPPSSPTFIHS  240

WT              PGYSPKRARKDSSSPTTTTPNFWSDK  266
```

Vgll4b E3(Δ4+6)

WT open reading frame is underlined. Aminoacids coded by another coding frame after the mutation site are in *red* italics.

```
WT               CTTTCTGCGATTAAAAGTCCATAAATGCTTTTTACCAAAATGGACCTGTTGAACTACCAG  180
                 ||||||||||||||||||||||||||||||||||||||||||||||||||||||||||||
Vgll4b E3Δ4+6    CTTTCTGCGATTAAAAGTCCATAAATGCTTTTTACCAAAATGGACCTGTTGAACTACCAG  180
                 L  S  A  I  K  S  P  *  M  L  F  T  K  M  D  L  L  N  Y  Q
                  F  L  R  L  K  V  H  K  C  F  L  P  K  W  T  C  *  T  T  S
                   F  C  D  *  K  S  I  N  A  F  Y  Q  N  G  P  V  E  L  P  V

WT               TACTTGGACAAAATGAACAACAACATCGGCATCCTGTGCTACGAAGGAGATGCGGCTCTG  240
                 ||||||||||||||||||||||||||||||||||||||||||||||||||||||||||||
Vgll4b E3Δ4+6    TACTTGGACAAAATGAACAACAACATCGGCATCCTGTGCTACGAAGGAGATGCGGCTCTG  240
                 Y  L  D  K  M  N  N  N  I  G  I  L  C  Y  E  G  D  A  A  L
                  T  W  T  K  *  T  T  T  S  A  S  C  A  T  K  E  M  R  L  *
                   L  G  Q  N  E  Q  Q  H  R  H  P  V  L  R  R  R  C  G  S  E

WT               AGAGGCGAGTCCAGAATGCAGTCTCTGTCGTCTGCAGTCAGCAACCACCGGACCGGCCCT  300
                 ||||||||||||||||||||||||||||||||||||||||||||||||||||||||||||
Vgll4b E3Δ4+6    AGAGGCGAGTCCAGAATGCAGTCTCTGTCGTCTGCAGTCAGCAACCACCGGACCGGCCCT  300
                 R  G  E  S  R  M  Q  S  L  S  S  A  V  S  N  H  R  T  G  P
                  E  A  S  P  E  C  S  L  C  R  L  Q  S  A  T  T  G  P  A  L
                   R  R  V  Q  N  A  V  S  V  V  C  S  Q  Q  P  P  D  R  P  S

WT               CCTCCCATCAGCCCCCAGCAAGAGGAAACACAGCGCGGAGCAAGCAGACGACGACATCGAC  360
                 ||||||||||||||||||||||||||||||||||||||||||||||||||||||||||||
Vgll4b E3Δ4+6    CCTCCCATCAGCCCCCAGCAAGAGGAAACACAGCGCGGAGCAAGCAGACGACGACATCGAC  360
                 P  P  I  S  P  S  K  R  K  H  S  A  E  Q  A  D  D  D  I  D
                  L  P  S  A  P  A  R  G  N  T  A  R  S  K  Q  T  T  T  S  T
                   S  H  Q  P  Q  Q  E  E  T  Q  R  G  A  S  R  R  R  H  R  L

WT               TGCAACAGTGAGCATGTGGCCAAGATGAGCCGCCTGTTCGCCGCACAGCTAGGAAAGCCT  420
                 ||||||||||||||||||||||||||||||||||||||||||||||||||||||||||||
Vgll4b E3Δ4+6    TGCAACAGTGAGCATGTGGCCAAGATGAGCCGCCTGTTCGCCGCACAGCTAGGAAAGCCT  420
                 C  N  S  E  H  V  A  K  M  S  R  L  F  A  A  Q  L  G  K  P
                  A  T  V  S  M  W  P  R  *  A  A  C  S  P  H  S  *  E  S  L
                   Q  Q  *  A  C  G  Q  D  E  P  P  V  R  R  T  A  R  K  A  C

WT               GCCAACGGGGACTACCGCAAGGACCCCCGGGAGCGCAGCCGCAGCCCCATCGAGC--GCA  478
                 |||||||||||||||||||||||| ||||||||||||||||||||||||||     |||
Vgll4b E3Δ4+6    GCCAACGGGGACTACCGCAAGGACCTCCGGGAGCGCAGCCGCAGCCCCATCATCCCAGCA  480
                 A  N  G  D  Y  R  K  D  L  R  E  R  S  R  S  P  I  I  P  A
                  P  T  G  T  T  A  R  T  S  G  S  A  A  A  A  P  S  S  Q  H
                   Q  R  G  L  P  Q  G  P  P  G  A  Q  P  Q  P  H  H  P  S  M

WT               TGGCGGCCCCCAGCATGAGTCTGGTTGGTGGGCATCATCTCTACATGCCCAGCCTGGCCC  538
                 ||||||||||||||||||||||||||||||||||||||||||||||||||||||||||||
Vgll4b E3Δ4+6    TGGCGGCCCCCAGCATGAGTCTGGTTGGTGGGCATCATCTCTACATGCCCAGCCTGGCCC  540
                 W  R  P  P  A  *  V  W  L  V  G  I  I  S  T  C  P  A  W  P
                  G  G  P  Q  H  E  S  G  W  W  A  S  S  L  H  A  Q  P  G  P
                   A  A  P  S  M  S  L  V  G  G  H  H  L  Y  M  P  S  L  A  L
```

Peptide

```
WT               MLFTKMDLLNYQYLDKMNNNIGILCYEGDAALRGESRMQSLSSAVSNHRTGPPPISPSKR   60
                 ||||||||||||||||||||||||||||||||||||||||||||||||||||||||||||
Vgll4b E3Δ4+6    MLFTKMDLLNYQYLDKMNNNIGILCYEGDAALRGESRMQSLSSAVSNHRTGPPPISPSKR   60

WT               KHSAEQADDDIDCNSEHVAKMSRLFAAQLGKPANGDYRKDPRERSRSPIERMAAPSMSLV  120
                 |||||||||||||||||||||||||||||||||||||||| ||||||||
Vgll4b E3Δ4+6    KHSAEQADDDIDCNSEHVAKMSRLFAAQLGKPANGDYRKDLRERSRSPIIPAWRPPA

WT               GGHHLYMPSLALDQPLALTKNMDSSRSMGISPTASPVERQQNRPSVITCAPANNRNCNLS  180

WT               HCTGSHNGCSPGLNASYRRASNSNTACDPVIEEHFRRSLGKNYKEPEPVTNSVSITGSVD  240

WT               DHFAKALGETWLQIKAKGGSSSPDASPNTHMVNHNHSPSLVS  282
```

Yap E3(Δ8)

WT open reading frame is underlined. Aminoacids coded by another coding frame after the mutation site are in *red* italics.

```
WT         TTCAGTTGGATAGAAAGAAGAGTTTTACTTTTATCTGGAGTCATGGATCCGAACCAGCACAACCCTCCAGCCGGCCACCA  440
           ||||||||||||||||||||||||||||||||||||||||||||||||||||||||||||||||||||||||||||||||
Yap E3Δ8   TTCAGTTGGATAGAAAGAAGAGTTTTACTTTTATCTGGAGTCATGGATCCGAACCAGCACAACCCTCCAGCCGGCCACCA  440
           F  S  W  I  E  R  R  V  L  L  L  S  G  V  M  D  P  N  Q  H  N  P  P  A  G  H  Q
            S  V  G  *  K  E  E  F  Y  F  Y  L  E  S  W  I  R  T  S  T  T  L  Q  P  A  T  R
             Q  L  D  R  K  K  S  F  T  F  I  W  S  H  G  S  E  P  A  Q  P  S  S  R  P  P

WT         GATCGTCCATGTTCGGGGAGACTCCGAGACCGATCTGGAGGCTCTTTTTAACGCTGTGATGAACCCGAAAAACACCATCG  520
           ||||||||||||||||||||||||||||||||||||||||||||||||||||||||||||||||||||||||||||||||
Yap E3Δ8   GATCGTCCATGTTCGGGGAGACTCCGAGACCGATCTGGAGGCTCTTTTTAACGCTGTGATGAACCCGAAAAACACCATCG  520
            I  V  H  V  R  G  D  S  E  T  D  L  E  A  L  F  N  A  V  M  N  P  K  N  T  I  V
             S  S  M  F  G  E  T  P  R  P  I  W  R  L  F  L  T  L  *  *  T  R  K  T  P  S
           D  R  P  C  S  G  R  L  R  D  R  S  G  G  S  F  *  R  C  D  E  P  E  K  H  H  R

WT         TCCCCCCTTCCGTGCCGATGAGGTTGAGAAAGCTGCCAGACTCATTCTTCACGCCGCCAGAGCCAAAGTCCCACTCCAGA  600
           ||||||||||||||||||||||||||||||||||||||||||||||||||||||||||||||||||||||||||||||||
Yap E3Δ8   TCCCCCCTTCCGTGCCGATGAGGTTGAGAAAGCTGCCAGACTCATTCTTCACGCCGCCAGAGCCAAAGTCCCACTCCAGA  600
             P  P  S  V  P  M  R  L  R  K  L  P  D  S  F  F  T  P  P  E  P  K  S  H  S  R
           S  P  L  P  C  R  *  G  *  E  S  C  Q  T  H  S  S  R  R  Q  S  Q  S  P  T  P  D
            P  P  F  R  A  D  E  V  E  K  A  A  R  L  I  L  H  A  A  R  A  K  V  P  L  Q  T

WT         CAAGCCAGTACAGATGCAGGTACTGCTGGTACCGTCACGCCCCATCACGTCCGGGCACACTCCTCACCTGCCTCCCTGCA  680
           ||||||||||||||||||||||||||||||||||||||||||||||||||||||||||||||||||||||||||||||||
Yap E3Δ8   CAAGCCAGTACAGATGCAGGTACTGCTGGTACCGTCACGCCCCATCACGTCCGGGCACACTCCTCACCTGCCTCCCTGCA  680
           Q  A  S  T  D  A  G  T  A  G  T  V  T  P  H  H  V  R  A  H  S  S  P  A  S  L  Q
            K  P  V  Q  M  Q  V  L  L  V  P  S  R  P  I  T  S  G  H  T  P  H  L  P  P  C  S
             S  Q  Y  R  C  R  Y  C  W  Y  R  H  A  P  S  R  P  G  T  L  L  T  C  L  P  A

WT         GCTGGGCGCAGTTTCTCCTGGTGCACTGACCAGCATGGGTCCAGCAAACGCCCCACCTCAGCACCTCCGCCAGTCCTCTT  760
           ||||||||||||||||||||||||||||||||||||||||||||||||||||||||||||||||||||||||||||||||
Yap E3Δ8   GCTGGGCGCAGTTTCTCCTGGTGCACTGACCAGCATGGGTCCAGCAAACGCCCCACCTCAGCACCTCCGCCAGTCCTCTT  760
            L  G  A  V  S  P  G  A  L  T  S  M  G  P  A  N  A  P  P  Q  H  L  R  Q  S  S  Y
             W  A  Q  F  L  L  V  H  *  P  A  W  V  Q  Q  T  P  H  L  S  T  S  A  S  P  L
           A  G  R  S  F  S  W  C  T  D  Q  H  G  S  S  K  R  P  T  S  A  P  P  P  V  L  L

WT         ACGAGATACCTGATGACATGCCGCTGCCCCCAGGCTGGGAGATGGCCAAGACCCCTTCGGGACAGAGATACTTCCTAAACCATAA  847
           |||||||||||||        ||||||||||||||||||||||||||||||||||||||||||||||||||||||||||||||||
Yap E3Δ8   ACGAGATACCTGA--------CGCTGCCCCCAGGCTGGGAGATGGCCAAGACCCCTTCGGGACAGAGATACTTCCTAAACCATAA
            E  I  P  D              A  A  P  R  L  G  D  G  Q  D  P  F  G  T  E  I  L  P  K  P  *
           T  R  Y  L  T                L  P  P  G  W  E  M  A  K  T  P  S  G  Q  R  Y  F  L  N  H  N
     R  D  T  *            R  C  P  Q  A  G  R  W  P  R  P  L  R  D  R  D  T  S  *  T  I  M
```

Peptide

```
WT         MDPNQHNPPAGHQIVHVRGDSETDLEALFNAVMNPKNTIVPPSVPMRLRKLPDSFFTPPE  60
           ||||||||||||||||||||||||||||||||||||||||||||||||||||||||||||
Yap E3Δ8   MDPNQHNPPAGHQIVHVRGDSETDLEALFNAVMNPKNTIVPPSVPMRLRKLPDSFFTPPE  60

WT         PKSHSRQASTDAGTAGTVTPHHVRAHSSPASLQLGAVSPGALTSMGPANAPPQHLRQSSY  120
           ||||||||||||||||||||||||||||||||||||||||||||||||||||||||||||
Yap E3Δ8   PKSHSRQASTDAGTAGTVTPHHVRAHSSPASLQLGAVSPGALTSMGPANAPPQHLRQSSY  120

WT         EIPDDMPLPPGWEMAKTPSGQRYFLNHNDQTTTWQDPRKALLQMNQAAPASPVPVQQQNI  180
           ||||
Yap E3Δ8   EIPDAAPRLGDGQDPFGTEILPKP

WT         MNPASGPLPDGWEQAITSEGEIYYINHKNKTTSWLDPRLDPRFAMNQQRISQSAPVKQGS  240

WT         QLPSSPQSGVMSGNNPIRLQQIHIEKERLRIKQELLRQRPQELALRNQLPTSMEQDGGTQ  300

WT         NPVSSPGMGQDARNMTTNSSDPFLNSGTYHSRDESTDSGLSMSSYSVPRTPDDFLNSVDE  360

WT         METGDTLGPGSMATQPSRFPDYLDAIPGTDVDLGTLEGESMAVEGEELMPSLQEALSSDI  420

WT         LNDMESVLAATKIDKENFLTWL  442
```

TAZ E1(Δ4)

WT open reading frame is underlined. Aminoacids coded by another coding frame after the mutation site are in *red* italics.

```
WT          CAAACACTTTTCAACTCGGCTGAAACTACTTAAGGACGAAAAACAGGAAAAGTTCCTACA  60
            ||||||||||||||||||||||||||||||||||||||||||||||||||||||||||||
Taz E1Δ4    CAAACACTTTTCAACTCGGCTGAAACTACTTAAGGACGAAAAACAGGAAAAGTTCCTACA  60
            Q  T  L  F  N  S  A  E  T  T  *  G  R  K  T  G  K  V  P  T
             K  H  F  S  T  R  L  K  L  L  K  D  E  K  Q  E  K  F  L  Q
              N  T  F  Q  L  G  *  N  Y  L  R  T  K  N  R  K  S  S  Y  R

WT          GGAAtttttttttCCATCGGCCATTTTAATCGAAGTTTGTTTTGACCATGAGCGGTAATC  120
            ||||||||||||||||||||||||||||||||||||||||||||||||||||||||||||
Taz E1Δ4    GGAATTTTTTTTTCCATCGGCCATTTTAATCGAAGTTTGTTTTGACCATGAGCGGTAATC  120
            G  I  F  F  S  I  G  H  F  N  R  S  L  F  *  P  *  A  V  I
             E  F  F  F  P  S  A  I  L  I  E  V  C  F  D  H  E  R  *  S
              N  F  F  F  H  R  P  F  *  S  K  F  V  L  T  M  S  G  N  P

WT          CTCTCCAGCCGATACCGGGCCACCAGGTGATCCATGTCGCCAAAGACCTGGACACGGATC  180
            ||||||||||||||||||||||||||||||||||||||||||||||||||||||||||||
Taz E1Δ4    CTCTCCAGCCGATACCGGGCCACCAGGTGATCCATGTCGCCAAAGACCTGGACACGGATC  180
            L  S  S  R  Y  R  A  T  R  *  S  M  S  P  K  T  W  T  R  I
             S  P  A  D  T  G  P  P  G  D  P  C  R  Q  R  P  G  H  G  S
              L  Q  P  I  P  G  H  Q  V  I  H  V  A  K  D  L  D  T  D  L

WT          TGGAGGCTCTTTTTAACTCGGTCATGAACCCGAAACCGAGCTCCTGGAGGAACAAGGATA  240
            ||||||||||||||||||||||||||||||||||||||||||||||||||||||||||||
Taz E1Δ4    TGGAGGCTCTTTTTAACTCGGTCATGAACCCGAAACCGAGCTCCTGGAGGAACAAGGATA  240
            W  R  L  F  L  T  R  S  *  T  R  N  R  A  P  G  G  T  R  I
             G  G  S  F  *  L  G  H  E  P  E  T  E  L  L  E  E  Q  G  Y
              E  A  L  F  N  S  V  M  N  P  K  P  S  S  W  R  N  K  D  M

WT          TGCCGCAGTCTTTCTTCCAGGAGCCGGACTCGGGCTCCCACTCCCGGCAGTCCAGCGCGG  300
            ||||||||||||||||||||||||||||||||||||||||||||||||||||||||||||
Taz E1Δ4    TGCCGCAGTCTTTCTTCCAGGAGCCGGACTCGGGCTCCCACTCCCGGCAGTCCAGCGCGG  300
            C  R  S  L  S  S  R  S  R  T  R  A  P  T  P  G  S  P  A  R
             A  A  V  F  L  P  G  A  G  L  G  L  P  L  P  A  V  Q  R  G
              P  Q  S  F  F  Q  E  P  D  S  G  S  H  S  R  Q  S  S  A  D

WT          ATTCCGGTTCTCTCCCGCCGAGGGTCCACTTTCGCTCGCGCTCATCTCCGGCGTCCCTAC  360
            ||||||||||||||||||||||||||||||||||||||||||||||||||||||||||||
Taz E1Δ4    ATTCCGGTTCTCTCCCGCCGAGGGTCCACTTTCGCTCGCGCTCATCTCCGGCGTCCCTAC  360
            I  P  V  L  S  R  R  G  S  T  F  A  R  A  H  L  R  R  P  Y
             F  R  F  S  P  A  E  G  P  L  S  L  A  L  I  S  G  V  P  T
              S  G  S  L  P  P  R  V  H  F  R  S  R  S  S  P  A  S  L  Q

WT          AGCTGCCGGCGGGCTCCGTGAGCGGCCCGAGCCCCGGGAGACTCCACTCCCACACCCGGC  420
            ||    ||||||||||||||||||||||||||||||||||||||||||||||||||||||
Taz E1Δ4    AG----CGGCGGGCTCCGTGAGCGGCCCGAGCCCCGGGAGACTCCACTCCCACACCCGGC  416
            S      G  G  L  R  E  R  P  E  P  R  E  T  P  L  P  H  P  A
             A       A  G  S  V  S  G  P  S  P  G  R  L  H  S  H  T  R  H
                  R  R  A  P  *  A  A  R  A  P  G  D  S  T  P  T  P  G
```

Peptide

```
WT          MSGNPLQPIPGHQVIHVAKDLDTDLEALFNSVMNPKPSSWRNKDMPQSFFQEPDSGSHSR  60
            ||||||||||||||||||||||||||||||||||||||||||||||||||||||||||||
TAZ E1Δ4    MSGNPLQPIPGHQVIHVAKDLDTDLEALFNSVMNPKPSSWRNKDMPQSFFQEPDSGSHSR  60

WT          QSSADSGSLPPRVHFRSRSSPASLQLPAGSVSGPSPGRLHSHTRHQSCDVAEELPLPPGW  120
            |||||||||||||||||||||||||
TAZ E1Δ4    QSSADSGSLPPRVHFRSRSSPASLQRRAP

WT          EMAFTPNGQKYFLNHIEKITTWHDPRKSMTPSVAQLSLHNQVSNTASIQQRSMALSQPNL  180

WT          VLNQQAHQQQQQHLQQQQQQVPVQVPVQAPQQQSSQPMMNLSAQQHQQKMRLQRIQMERE  240

WT          RIQRRQEELMRQEVALRQLPMDSDNLPPVAPAIGSPAMSAGNMPNNSADPFLNSGPYHSR  300

WT          EQSTDSGLGLGCYSIPTTPEDFLNNMEDMDTGENMVPVSMNVPPQSRFPDFLDSMPGTNV  360

WT          DLGTLEGTDLMPILNDVESVLNKSEPFLTWL 391
```

Tead3a[SA14593]

WT open reading frame is underlined. Aminoacids coded by another coding frame after the mutation site are in *red* italics.

```
WT                CCAGCGAGAGCCCGGAGGAGGGCCAGGAGGATGAGGTGGACGGGATGGACAAAACCGGAA  360
                  ||||||||||||||||||||||||||||||||||||||||||||||||||||||||||||
Tead3a SA14593    CCAGCGAGAGCCCGGAGGAGGGCCAGGAGGATGAGGTGGACGGGATGGACAAAACCGGAA  360
                  P  A  R  A  R  R  R  A  R  R  M  R  W  T  G  W  T  K  P  E
                   Q  R  E  P  G  G  G  P  G  G  *  G  G  R  D  G  Q  N  R  N
                    S  E  S  P  E  E  G  Q  E  D  E  V  D  G  M  D  K  T  G  M

WT                TGGACGGGGATGCGGAGGGCGTCTGGAGTCCTGACATTGAACAAAGCTTCCAGGAGGCTC  420
                  ||||||||||||||||||||||||||||||||||||||||||||||||||||||||||||
Tead3a SA14593    TGGACGGGGATGCGGAGGGCGTCTGGAGTCCTGACATTGAACAAAGCTTCCAGGAGGCTC  420
                  W  T  G  M  R  R  A  S  G  V  L  T  L  N  K  A  S  R  R  L
                   G  R  G  C  G  G  R  L  E  S  *  H  *  T  K  L  P  G  G  S
                    D  G  D  A  E  G  V  W  S  P  D  I  E  Q  S  F  Q  E  A  L

WT                TGGCCATTTATCCACCCTGTGGCCGCAGAAAGATCATCCTCTCAGATGAAGGAAAGATGT  480
                  ||||||||||||||||||||||||||||||||||||||||||||||||||||||||||||
Tead3a SA14593    TGGCCATTTATCCACCCTGTGGCCGCAGAAAGATCATCCTCTCAGATGAAGGAAAGATGT  480
                  W  P  F  I  H  P  V  A  A  E  R  S  S  S  Q  M  K  E  R  C
                   G  H  L  S  T  L  W  P  Q  K  D  H  P  L  R  *  R  K  D  V
                    A  I  Y  P  P  C  G  R  R  K  I  I  L  S  D  E  G  K  M  Y

WT                ATGGTCGTAATGAATTGATAGCAAGATATATAAAGCTGCGAACTGGAAAAACACGCACAC  540
                  ||||||||||||||||||||||||||||||||||||||||||||||||||||||||||||
Tead3a SA14593    ATGGTCGTAATGAATTGATAGCAAGATATATAAAGCTGCGAACTGGAAAAACACGCACAC  540
                  M  V  V  M  N  *  *  Q  D  I  *  S  C  E  L  E  K  H  A  H
                   W  S  *  *  I  D  S  K  I  Y  K  A  A  N  W  K  N  T  H  T
                    G  R  N  E  L  I  A  R  Y  I  K  L  R  T  G  K  T  R  T  R

WT                GGAAACAGGTATCTAGTCACATACAGGTGTTAGCAAGGAAGAAAATGCGGGAATATCAAG  600
                  ||||||||||||||||||||||||||||||||||||||||||||||||||||||||||||
Tead3a SA14593    GGAAACAGGTATCTAGTCACATACAGGTGTTAGCAAGGAAGAAAATGCGGGAATATCAAG  600
                  G  N  R  Y  L  V  T  Y  R  C  *  Q  G  R  K  C  G  N  I  K
                   E  T  G  I  *  S  H  T  G  V  S  K  E  E  N  A  G  I  S  S
                    K  Q  V  S  S  H  I  Q  V  L  A  R  K  K  M  R  E  Y  Q  A

WT                CAGGCATAAAGGCCATGAATTTGGATCAGGCTTCAAAAGACAAAGCTCTTCAGAACATGG  660
                  ||||||||||||||||||||||||||||||||||||||||||||||||||||||||||||
Tead3a SA14593    CAGGCATAAAGGCCATGAATTTGGATCAGGCTTCAAAAGACAAAGCTCTTCAGAACATGG  660
                  Q  A  *  R  P  *  I  W  I  R  L  Q  K  T  K  L  F  R  T  W
                   R  H  K  G  H  E  F  G  S  G  F  K  R  Q  S  S  S  E  H  G
                    G  I  K  A  M  N  L  D  Q  A  S  K  D  K  A  L  Q  N  M  A

WT                CGGCTCTGTCCTCAGCTCAGATCGTGTCTGCCAGTGTGATGAAGAGTCAGCTTCCTCCTC  720
                  ||||||||||||||||||||||||||||||||||||||||||||||||||||||||||||
Tead3a SA14593    CGGCTCTGTCCTCAGCTCAGATCGTGTCTGCCAGTGTGATGAAGAGTCAGCTTCCTCCTC  720
                  R  L  C  P  Q  L  R  S  C  L  P  V  *  *  R  V  S  F  L  L
                   G  S  V  L  S  S  D  R  V  C  Q  C  D  E  E  S  A  S  S  S
                    A  L  S  S  A  Q  I  V  S  A  S  V  M  K  S  Q  L  P  P  L

WT                TTCCTCAGCACCCATACCCTCCTCCAGCCCGGTTTTGGCCCGGCCCCATCCCAGGACAGC  780
                  ||||||||||||||||||||||||||||||||||||||||||||||||||||||||||||
Tead3a SA14593    TTCCTCAGCACCCATACCCTCCTCCAGCCCGGTTTTGGCCCGGCCCCATCCCAGGACAGC  780
                  F  L  S  T  H  T  L  L  Q  P  G  F  G  P  A  P  S  Q  D  S
                   S  S  A  P  I  P  S  S  S  P  V  L  A  R  P  H  P  R  T  A
                    P  Q  H  P  Y  P  P  P  A  R  F  W  P  G  P  I  P  G  Q  P

WT                CTGGACCTTCTCAGGACATCAAGCCCTTTGCACCGAGCCCGTACCCTACCCTTCAGCCTC  840
                  ||||||||||||||||
Tead3a SA14593    CTGGACCTTCTCAGGAggagtatttcagacccacatttgatctaagaacagtactcctgc  840
                  L  D  L  L  R  R  E  Y  F  R  P  T  F  D  L  R  T  V  L  L
                   W  T  F  S  G  G  S  I  S  D  P  H  L  I  *  E  Q  Y  S  C
```

G P S Q *E G V F Q T H I ** S K N S T P A

Tead3a^{SA14593} Peptide

```
WT               MDKTGMDGDAEGVWSPDIEQSFQEALAIYPPCGRRKIILSDEGKMYGRNELIARYIKLRT  60
                 ||||||||||||||||||||||||||||||||||||||||||||||||||||||||||||
Tead3a SA14593   MDKTGMDGDAEGVWSPDIEQSFQEALAIYPPCGRRKIILSDEGKMYGRNELIARYIKLRT  60

WT               GKTRTRKQVSSHIQVLARKKMREYQAGIKAMNLDQASKDKALQNMAALSSAQIVSASVMK  120
                 ||||||||||||||||||||||||||||||||||||||||||||||||||||||||||||
Tead3a SA14593   GKTRTRKQVSSHIQVLARKKMREYQAGIKAMNLDQASKDKALQNMAALSSAQIVSASVMK  120

WT               SQLPPLPQHPYPPPARFWPGPIPGQPGPSQDIKPFAPSPYPTLQPPPISSYEQLPAPLPP  180
                 ||||||||||||||||||||||||||||||
Tead3a SA14593   SQLPPLPQHPYPPPARFWPGPIPGQPGPSQEGVFQTHI

WT               SATAVPVWQDRTIASSKLRMLEYSAFMEVQRDPDTYSKHLFVHIAQTNPSYTDPLLEAVD  240

WT               IRQIYDKFPEKKGGLKELYEKGPQNAFFLVKFWADLNSSNVQDGAGSFYGVSSQYSSAEN  300

WT               MTITVSTKVCSFGKQVVEKVETEYARVEGGRYVYRIHRSPMCEYMINFIHKLKHLPEKYM  360

WT               MNSVLENFTILQVVTNRDTQETLLCIAFVFEVSTSDHGAQYHVYRLVKD 409
```
