# Supplementary material for: TEADs, Yap, Taz, Vgll4s transcription factors control the establishment of Left-Right asymmetry in zebrafish
Source: eLife. 2019 Sep 12;8:e45241. doi: 10.7554/eLife.45241 (PMC6759317; doi:10.7554/eLife.45241)

Supplementary file 8: Position of MO target sequences and mutations (red) in dnmt3bb.1, dnmt3bb.2, dnmt3ba, mbd3a and mbd3b

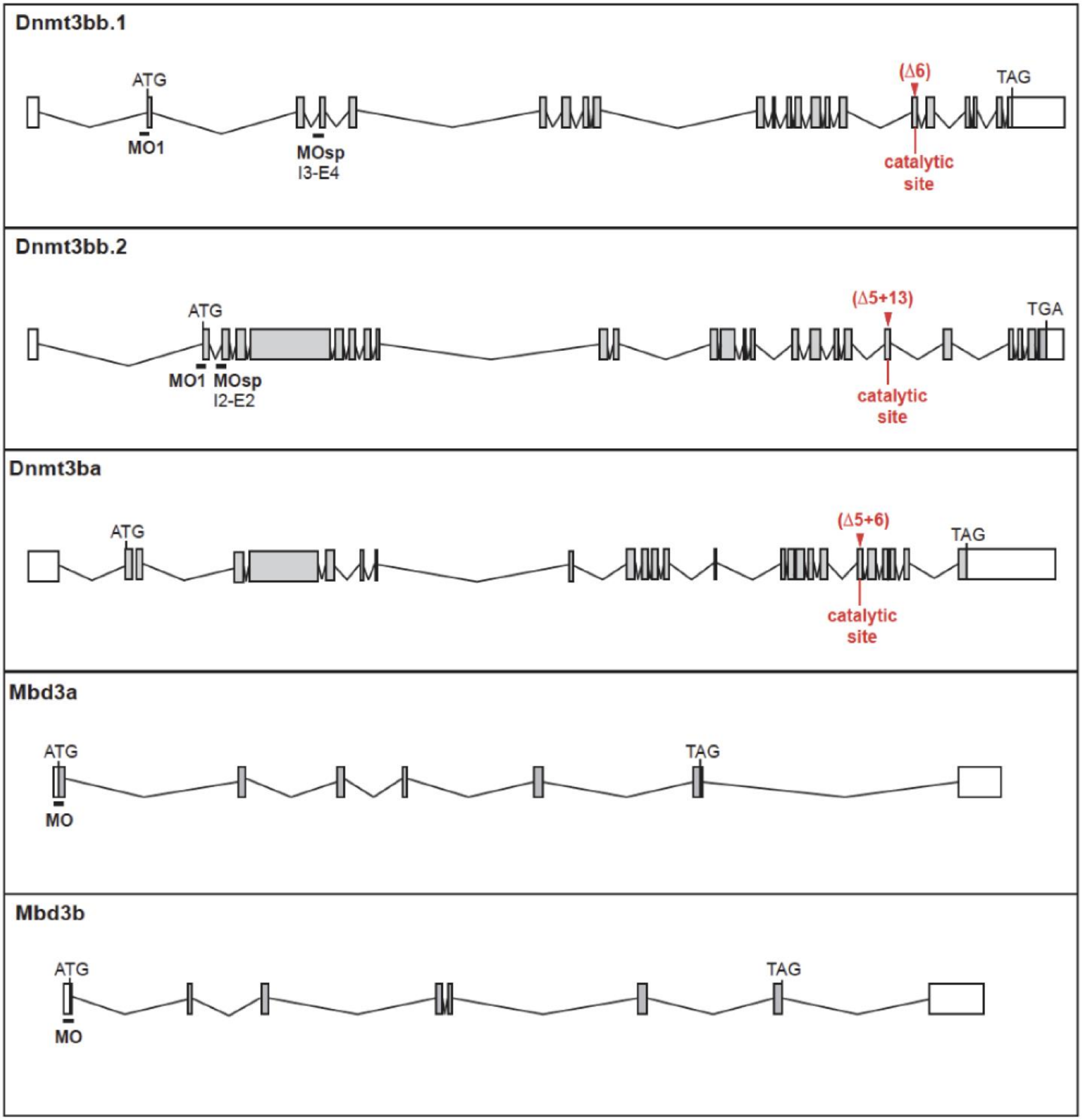

Supplement: Supplementary file 8. [file elife-45241-supp8.pdf]
