# Supplementary material for: TEADs, Yap, Taz, Vgll4s transcription factors control the establishment of Left-Right asymmetry in zebrafish
Source: eLife. 2019 Sep 12;8:e45241. doi: 10.7554/eLife.45241 (PMC6759317; doi:10.7554/eLife.45241)
Supplement: Supplementary file 9. [file elife-45241-supp9.pdf]

Supplementary File 9: Sequence of Dnmt3bb.1, Dnmt3bb.2 and Dnmt3ba mutants. Catalytic site is underlined. TALEN target sequences are in green. Aminoacids coded on the wrong reading frame after the mutation site are italicized and red.

DNMT3BB.1 E17(Δ6)

```
                   W  G  P  F  D  M  V  I  G  G  S  P  C  N  D  L  S  I  V  N  P  A  R
Dnmt3bb.1          TGGGGGCCCTTTGACATGGTGATTGGTGGAAGTCCCTGTAATGACCTTTCAATTGTCAACCCTGCTAGG 1940
                   ||||||||||||||||||||||||||||||      |||||||||||||||||||||||||||||||||
Dnmt3bb.1(Δ6)      TGGGGGCCCTTTGACATGGTGATTGGTGGA------TGTAATGACCTTTCAATTGTCAACCCTGCTAGG 1934
                   W  G  P  F  D  M  V  I  G  G        C  N  D  L  S  I  V  N  P  A  R
```

```
Dnmt3bb.1        MRKEEIKKSSEIVMPSNKYPSAESDKMTATAAMNRDTSVGDGLSENDSGLEMTSENSPLT  60
                 ||||||||||||||||||||||||||||||||||||||||||||||||||||||||||||
Dnmt3bb.1(Δ6)    MRKEEIKKSSEIVMPSNKYPSAESDKMTATAAMNRDTSVGDGLSENDSGLEMTSENSPLT  60

Dnmt3bb.1        PAEPPSPFCPKQNGGAASPADESVNSIRRKRSRKRSDTEEDSAWDSSNSEEKAEVGSGCE  120
                 ||||||||||||||||||||||||||||||||||||||||||||||||||||||||||||
Dnmt3bb.1(Δ6)    PAEPPSPFCPKQNGGAASPADESVNSIRRKRSRKRSDTEEDSAWDSSNSEEKAEVGSGCE  120

Dnmt3bb.1        TGLRQRPRPRTIFQAGLTAHSKPRSRERGHSKEDHSDLVASVPEGPALELMEQDSKDSAQ  180
                 ||||||||||||||||||||||||||||||||||||||||||||||||||||||||||||
Dnmt3bb.1(Δ6)    TGLRQRPRPRTIFQAGLTAHSKPRSRERGHSKEDHSDLVASVPEGPALELMEQDSKDSAQ  180

Dnmt3bb.1        SSTTSTSTTETASQPEYKDNKGFGIGELVWGKIKGFSWWPGMVVTWRATGRRQASHGMRW  240
                 ||||||||||||||||||||||||||||||||||||||||||||||||||||||||||||
Dnmt3bb.1(Δ6)    SSTTSTSTTETASQPEYKDNKGFGIGELVWGKIKGFSWWPGMVVTWRATGRRQASHGMRW  240

Dnmt3bb.1        LQWFGDGKFSEVSADKLDSITAFPKFFNQSSYTKLASYRRAIFQALEVASLRAEKTFPPS  300
                 ||||||||||||||||||||||||||||||||||||||||||||||||||||||||||||
Dnmt3bb.1(Δ6)    LQWFGDGKFSEVSADKLDSITAFPKFFNQSSYTKLASYRRAIFQALEVASLRAEKTFPPS  300

Dnmt3bb.1        EADSLEEQVKPMLDWAHGGFLPKGQEGLKPKENAEYCVFPLASESSTLLESSPPEFPPSA  360
                 ||||||||||||||||||||||||||||||||||||||||||||||||||||||||||||
Dnmt3bb.1(Δ6)    EADSLEEQVKPMLDWAHGGFLPKGQEGLKPKENAEYCVFPLASESSTLLESSPPEFPPSA  360

Dnmt3bb.1        KRARLPLNKAKPGIEEVYSREQMVNEVLKNHRSIEEFCLSCGKTRVATFHPLFEGGLCLT  420
                 ||||||||||||||||||||||||||||||||||||||||||||||||||||||||||||
Dnmt3bb.1(Δ6)    KRARLPLNKAKPGIEEVYSREQMVNEVLKNHRSIEEFCLSCGKTRVATFHPLFEGGLCLT  420

Dnmt3bb.1        CKDAYLENSYMYDDDGYQSYCTVCCGGREMLLCGNANCCRCICVDCLDILVGAGAANSAR  480
                 ||||||||||||||||||||||||||||||||||||||||||||||||||||||||||||
Dnmt3bb.1(Δ6)    CKDAYLENSYMYDDDGYQSYCTVCCGGREMLLCGNANCCRCICVDCLDILVGAGAANSAR  480

Dnmt3bb.1        NLDPWRCYMCQPLQQYGVLKKRHDWSLKLQEFFVNDSGQEFESPKIYPAVPAEQRRPIRV  540
                 ||||||||||||||||||||||||||||||||||||||||||||||||||||||||||||
Dnmt3bb.1(Δ6)    NLDPWRCYMCQPLQQYGVLKKRHDWSLKLQEFFVNDSGQEFESPKIYPAVPAEQRRPIRV  540

Dnmt3bb.1        LSLFDGIATGYLVLRDLGFKVDLYIASEVCEDSISVGAVRHEGKIQYVHDVRNITRKNIA  600
                 ||||||||||||||||||||||||||||||||||||||||||||||||||||||||||||
Dnmt3bb.1(Δ6)    LSLFDGIATGYLVLRDLGFKVDLYIASEVCEDSISVGAVRHEGKIQYVHDVRNITRKNIA  600

Dnmt3bb.1        EWGPFDMVIGGSPCNDLSIVNPARKGLYEGTGRLFFEFYRLLSEAKPKEGEDRPFFWMFE  660
                 |||||||||||  |||||||||||||||||||||||||||||||||||||||||||||||
Dnmt3bb.1(Δ6)    EWGPFDMVIGG--CNDLSIVNPARKGLYEGTGRLFFEFYRLLSEAKPKEGEDRPFFWMFE  660

Dnmt3bb.1        NVVAMSVNDKRDISRFLECNPVMIDAIEVSAAHRARYFWGNLPGMKRPLCASGMDKLELQ  720
                 ||||||||||||||||||||||||||||||||||||||||||||||||||||||||||||
Dnmt3bb.1(Δ6)    NVVAMSVNDKRDISRFLECNPVMIDAIEVSAAHRARYFWGNLPGMKRPLCASGMDKLELQ  720

Dnmt3bb.1        DCLEHGRVAKFGKVRTITTRSNSIKQGKDQHFPVMMNGKEDILWCTELERIFGFPVHYTD  780
                 ||||||||||||||||||||||||||||||||||||||||||||||||||||||||||||
Dnmt3bb.1(Δ6)    DCLEHGRVAKFGKVRTITTRSNSIKQGKDQHFPVMMNGKEDILWCTELERIFGFPVHYTD  780

Dnmt3bb.1        VSNMGRGARQKLLGRSWSVPVIRHLFAPLKDYFACE  816
                 ||||||||||||||||||||||||||||||||||||
Dnmt3bb.1(Δ6)    VSNMGRGARQKLLGRSWSVPVIRHLFAPLKDYFACE  816
```

DNMT3BB.2 E20(Δ5+13)

```
                        W  G  P  F  D  L  L  I  G  G              S  P  C  N  D  L  S  I  V  N  P  A  R
Dnmt3bb.2               TGGGGTCCATTTGATCTTCTCATTGGTGGA-------------AGTCCATGTAATGACTTGTCCATAGTCAATCCTGCTCGG 3949
                        ||||||||||||||||||||||||||||||             |     |||||||||||||||||||||||||||||||||
Dnmt3bb.2 (Δ5+13)       TGGGGTCCATTTGATCTTCTCATTGGTGGAGAAGATCAATGTAA-----TGTAATGACTTGTCCATAGTCAATCCTGCTCGG 3957
                        W  G  P  F  D  L  L  I  G  G  E  D  Q  C  N     V  M  T  C  P  *
```

```
Dnmt3bb.2          MVADVKIGDDKQSLCELLDWLNGLLQATFSQVEDTCSGAAFCQLMDIIQPGSIDVTKVNF  60
                   ||||||||||||||||||||||||||||||||||||||||||||||||||||||||||||
Dnmt3bb.2(Δ5+13)   MVADVKIGDDKQSLCELLDWLNGLLQATFSQVEDTCSGAAFCQLMDIIQPGSIDVTKVNF  60

Dnmt3bb.2          TAEENLDILNNYNLLQEAFSKAQIQKELELTLLVNGDIMTTCDLLTWFKDMYDHNFAKQK  120
                   ||||||||||||||||||||||||||||||||||||||||||||||||||||||||||||
Dnmt3bb.2(Δ5+13)   TAEENLDILNNYNLLQEAFSKAQIQKELELTLLVNGDIMTTCDLLTWFKDMYDHNFAKQK  120

Dnmt3bb.2          CNPQVAFIKPEVVSLKSSREFETIEKENVSSLYNTEETSSNQKTQHVEKTSQESVSWSPL  180
                   ||||||||||||||||||||||||||||||||||||||||||||||||||||||||||||
Dnmt3bb.2(Δ5+13)   CNPQVAFIKPEVVSLKSSREFETIEKENVSSLYNTEETSSNQKTQHVEKTSQESVSWSPL  180

Dnmt3bb.2          TSFIRKYGSSTLTDDESNNVNSKDCPGQKSFGDITPFWRQTPYCLYLLHGVELEDDKKAS  240
                   ||||||||||||||||||||||||||||||||||||||||||||||||||||||||||||
Dnmt3bb.2(Δ5+13)   TSFIRKYGSSTLTDDESNNVNSKDCPGQKSFGDITPFWRQTPYCLYLLHGVELEDDKKAS  240

Dnmt3bb.2          VLLLGFFDKETGENKIRLLDVVYPTKESTEDICNYILDTLRKIGIPLFNMAILYSDFPDH  300
                   ||||||||||||||||||||||||||||||||||||||||||||||||||||||||||||
Dnmt3bb.2(Δ5+13)   VLLLGFFDKETGENKIRLLDVVYPTKESTEDICNYILDTLRKIGIPLFNMAILYSDFPDH  300

Dnmt3bb.2          EHLVAGLQLMKAEIVSLCGLTDLTGQVCHSGVEKIEFSDLILNLITEIYKHFPSFPADLQ  360
                   ||||||||||||||||||||||||||||||||||||||||||||||||||||||||||||
Dnmt3bb.2(Δ5+13)   EHLVAGLQLMKAEIVSLCGLTDLTGQVCHSGVEKIEFSDLILNLITEIYKHFPSFPADLQ  360

Dnmt3bb.2          ALLEDVVGSDIDNLTSQCSLFWRIIKKIPLAWSHLENYFGSLGTEEEAVCLLLEDPKIKL  420
                   ||||||||||||||||||||||||||||||||||||||||||||||||||||||||||||
Dnmt3bb.2(Δ5+13)   ALLEDVVGSDIDNLTSQCSLFWRIIKKIPLAWSHLENYFGSLGTEEEAVCLLLEDPKIKL  420

Dnmt3bb.2          NVLFLTHALQPLCDFQEIIDQGASVLQLLQDASKLLRLYTQSFLRPKAAEYFHRRGKTSL  480
                   ||||||||||||||||||||||||||||||||||||||||||||||||||||||||||||
Dnmt3bb.2(Δ5+13)   NVLFLTHALQPLCDFQEIIDQGASVLQLLQDASKLLRLYTQSFLRPKAAEYFHRRGKTSL  480

Dnmt3bb.2          VQETVGHLPRGEVAVGEQAADFLQQHSEELSDYLETFHSSIISFYTTVTVNIVKRLPLPD  540
                   ||||||||||||||||||||||||||||||||||||||||||||||||||||||||||||
Dnmt3bb.2(Δ5+13)   VQETVGHLPRGEVAVGEQAADFLQQHSEELSDYLETFHSSIISFYTTVTVNIVKRLPLPD  540

Dnmt3bb.2          STLRNLSLVLSPGKKLEVTGKMVQDLGVGFGVCIRPDNVSLLTDEFLEYQLIDGGDTGSV  600
                   ||||||||||||||||||||||||||||||||||||||||||||||||||||||||||||
Dnmt3bb.2(Δ5+13)   STLRNLSLVLSPGKKLEVTGKMVQDLGVGFGVCIRPDNVSLLTDEFLEYQLIDGGDTGSV  600

Dnmt3bb.2          DQPTEKYWQTELRIMGNASNFGKLIVSLLALPKTLKKEIIFKQMFQQTDYLKMMRKEDCE  660
                   ||||||||||||||||||||||||||||||||||||||||||||||||||||||||||||
Dnmt3bb.2(Δ5+13)   DQPTEKYWQTELRIMGNASNFGKLIVSLLALPKTLKKEIIFKQMFQQTDYLKMMRKEDCE  660

Dnmt3bb.2          EKDMMEDDVTDSSSYKSAPSHLSPETQGSSISDVIDLTEMDEIGPVEIEDIAPMDVDDIV  720
                   ||||||||||||||||||||||||||||||||||||||||||||||||||||||||||||
Dnmt3bb.2(Δ5+13)   EKDMMEDDVTDSSSYKSAPSHLSPETQGSSISDVIDLTEMDEIGPVEIEDIAPMDVDDIV  720

Dnmt3bb.2          SISSDSETENQKVNVPHVSIVLDDDDDDDDEMTDDDDDYGCEAGEVMWKYSKNKGNTQNE  780
                   ||||||||||||||||||||||||||||||||||||||||||||||||||||||||||||
Dnmt3bb.2(Δ5+13)   SISSDSETENQKVNVPHVSIVLDDDDDDDDEMTDDDDDYGCEAGEVMWKYSKNKGNTQNE  780

Dnmt3bb.2          MTDNTYQGGFSVGEMVLGPIEGFGLWPGLVQSWDSERPCGSMRKVIFFGNGMQTEVQADS  840
                   ||||||||||||||||||||||||||||||||||||||||||||||||||||||||||||
Dnmt3bb.2(Δ5+13)   MTDNTYQGGFSVGEMVLGPIEGFGLWPGLVQSWDSERPCGSMRKVIFFGNGMQTEVQADS  840

Dnmt3bb.2          LLPFSSLAKCFCSNSFATVMAYKDAIFSSLQVASRRSRMFFSPESESKDELLRVMLNWAF  900
                   ||||||||||||||||||||||||||||||||||||||||||||||||||||||||||||
Dnmt3bb.2(Δ5+13)   LLPFSSLAKCFCSNSFATVMAYKDAIFSSLQVASRRSRMFFSPESESKDELLRVMLNWAF  900

Dnmt3bb.2          GGFEPLGADGLQPQAEYSVKVKKGKRKNPTGKLFNLTVPLNKIPESLDLNNGSVDLGTTD  960
```

```
                          ||||||||||||||||||||||||||||||||||||||||||||||||||||||||||||
Dnmt3bb.2(Δ5+13)          GGFEPLGADGLQPQAEYSVKVKKGKRKNPTGKLFNLTVPLNKIPESLDLNNGSVDLGTTD  960

Dnmt3bb.2                 ADKKRLYSKWNGRSMQTVKIRRKYKQRNKNIIPTVQIESRQNSQKRHQMVHEFLKNKRKI  1020
                          ||||||||||||||||||||||||||||||||||||||||||||||||||||||||||||
Dnmt3bb.2(Δ5+13)          ADKKRLYSKWNGRSMQTVKIRRKYKQRNKNIIPTVQIESRQNSQKRHQMVHEFLKNKRKI  1020

Dnmt3bb.2                 EDFCLSCGSMSVDIIHPLFEGKLCTNCKFNFTETLYRYDEDGYQSYCTVCCSGMEVILCG  1080
                          ||||||||||||||||||||||||||||||||||||||||||||||||||||||||||||
Dnmt3bb.2(Δ5+13)          EDFCLSCGSMSVDIIHPLFEGKLCTNCKFNFTETLYRYDEDGYQSYCTVCCSGMEVILCG  1080

Dnmt3bb.2                 HDSCCRSFCVDCLDILVCQGTFDQLKNVDPWTCYLCAPETSSGALKPRHDWSIRVQEFFA  1140
                          ||||||||||||||||||||||||||||||||||||||||||||||||||||||||||||
Dnmt3bb.2(Δ5+13)          HDSCCRSFCVDCLDILVCQGTFDQLKNVDPWTCYLCAPETSSGALKPRHDWSIRVQEFFA  1140

Dnmt3bb.2                 NDTGMEFEPHRVYPSIPAIQRRPIRVLSLFDGIATGYLVLRDLGFKVEKYVASEIDEESI  1200
                          ||||||||||||||||||||||||||||||||||||||||||||||||||||||||||||
Dnmt3bb.2(Δ5+13)          NDTGMEFEPHRVYPSIPAIQRRPIRVLSLFDGIATGYLVLRDLGFKVEKYVASEIDEESI  1200

Dnmt3bb.2                 TISMVNHDGKITQVDDVKNITKKHIEQWGPFDLLIGGSPCNDLSIVNPARKGLYEGTGRL  1260
                          |||||||||||||||||||||||||||||||||||||
Dnmt3bb.2(Δ5+13)          TISMVNHDGKITQVDDVKNITKKHIEQWGPFDLLIGGEDCNVMTCP                1246

Dnmt3bb.2                 FFEYYRLLNVLKPKEDDPQPFFWLFENVTFMQTHVKADICRFLECNPVLVDAVKVSPAHR  1320

Dnmt3bb.2                 ARYFWGNIPGMNRPIIASQNDKLCLQECLEPGRTAKYEKVRTITTRQNSLKQGTNDAHFP  1380

Dnmt3bb.2                 VTMNGKDDHIWITELEKIFGFPKHYTDVKSMGRPQRQRVLGKSWSVPVIRHLLAPLKDYF  1440

Dnmt3bb.2                 ACDEFPVK  1448
```

DNMT3Ba E20(Δ5+6)

```
                    G  P  F  D  L  L  I  G  G  S  P  C  N  D              L  S  M  V  N  P  A  R  K  G
Dnmt3ba             GGGCCATTTGACCTTCTGATTGGTGGAAGTCCTTGTAATG------ACTTGTCCATGGTGAATCCAGCCAGAAAAGGT  3839
                    ||||||||||||||||||||||||||||||||||||||||           |||||||||||||||||||||||||||
Dnmt3ba (Δ5+6)      GGGCCATTTGACCTTCTGATTGGTGGAAGTCCTTGTAATGTCATGG-----TCCATGGTGAATCCAGCCAGAAAAGGT  3840
                    G  P  F  D  L  L  I  G  G  S  P  C  N  V  M  V        H  G  E  S  S  Q  K  R
```

```
Dnmt3ba       MATNVSLEPNNPDDKCSRYEVLGWINETLQTNFTQVEQCRSGACFCQLIDLLFPGTINLK  60
              ||||||||||||||||||||||||||||||||||||||||||||||||||||||||||||
Dnmt3ba1      MATNVSLEPNNPDDKCSRYEVLGWINETLQTNFTQVEQCRSGACFCQLIDLLFPGTINLK  60

Dnmt3ba       KVKFESQKRSDFMQNYGLLQAAFRDLEVTEPVPVNELLSGKFRPNFTYLKWFKKFFYANV  120
              ||||||||||||||||||||||||||||||||||||||||||||||||||||||||||||
Dnmt3ba       KVKFESQKRSDFMQNYGLLQAAFRDLEVTEPVPVNELLSGKFRPNFTYLKWFKKFFYANV  120

Dnmt3ba       KQERVYNAFEARDGQEIVPVDDVMKSPKALKSSYESGRAGEESDMEINGGRRSATYDPKW  180
              ||||||||||||||||||||||||||||||||||||||||||||||||||||||||||||
Dnmt3ba       KQERVYNAFEARDGQEIVPVDDVMKSPKALKSSYESGRAGEESDMEINGGRRSATYDPKW  180

Dnmt3ba       QRNLKWIRASDMGDNYAYCTTCDYNIILLAGFHDLKRHQLTQNHMKHETGRTNLPGRKQI  240
              ||||||||||||||||||||||||||||||||||||||||||||||||||||||||||||
Dnmt3ba       QRNLKWIRASDMGDNYAYCTTCDYNIILLAGFHDLKRHQLTQNHMKHETGRTNLPGRKQI  240

Dnmt3ba       EESISCSETMLLFIQSHCLSSLPSRINRVSQRTARCILGLKYPNDIVSACKLNPYCIYIY  300
              ||||||||||||||||||||||||||||||||||||||||||||||||||||||||||||
Dnmt3ba       EESISCSETMLLFIQSHCLSSLPSRINRVSQRTARCILGLKYPNDIVSACKLNPYCIYIY  300

Dnmt3ba       GQVPLDVKTGDKTNCHVVLAGFFEEKQARYCIRFLDVFQPEDSASSVSGGLFSILKKFEI  360
              ||||||||||||||||||||||||||||||||||||||||||||||||||||||||||||
Dnmt3ba       GQVPLDVKTGDKTNCHVVLAGFFEEKQARYCIRFLDVFQPEDSASSVSGGLFSILKKFEI  360

Dnmt3ba       PASNMVAVYINDHELTSESVVSQIRELNPQVIDLGGLYSIPDTACSAGLQTHSVQVQELI  420
              ||||||||||||||||||||||||||||||||||||||||||||||||||||||||||||
Dnmt3ba       PASNMVAVYINDHELTSESVVSQIRELNPQVIDLGGLYSIPDTACSAGLQTHSVQVQELI  420

Dnmt3ba       ANIYRHFSTGSTSNDNLKMLFAGIDGLKVHSNPLSNSEEFCVLVKRIHEMWSDLVSYFSS  480
              ||||||||||||||||||||||||||||||||||||||||||||||||||||||||||||
Dnmt3ba       ANIYRHFSTGSTSNDNLKMLFAGIDGLKVHSNPLSNSEEFCVLVKRIHEMWSDLVSYFSS  480

Dnmt3ba       CDENNDNVKQICSQLENPKIRLTLMFLDQALGPLRAFGQHLQQSKSSVRADLVEILREAS  540
              ||||||||||||||||||||||||||||||||||||||||||||||||||||||||||||
Dnmt3ba       CDENNDNVKQICSQLENPKIRLTLMFLDQALGPLRAFGQHLQQSKSSVRADLVEILREAS  540

Dnmt3ba       GLLRSYASSFLRPQAVIRYLKEQDPAILDNEAFCLPAAELSLGGVLEDFISAREEELADF  600
              ||||||||||||||||||||||||||||||||||||||||||||||||||||||||||||
Dnmt3ba       GLLRSYASSFLRPQAVIRYLKEQDPAILDNEAFCLPAAELSLGGVLEDFISAREEELADF  600

Dnmt3ba       LSTFYNECLAIYKTLTTSIAASLPLSDSVLRAISQLLSPAGRLKVTGKNIVDLAVRFGFC  660
              ||||||||||||||||||||||||||||||||||||||||||||||||||||||||||||
Dnmt3ba       LSTFYNECLAIYKTLTTSIAASLPLSDSVLRAISQLLSPAGRLKVTGKNIVDLAVRFGFC  660

Dnmt3ba       SKPEDSAKLNDEFLEYQLAEEENLSSTHSIERYWCTVLKTFPPTSVFKRLVLCLLVLPSP  720
              ||||||||||||||||||||||||||||||||||||||||||||||||||||||||||||
Dnmt3ba       SKPEDSAKLNDEFLEYQLAEEENLSSTHSIERYWCTVLKTFPPTSVFKRLVLCLLVLPSP  720

Dnmt3ba       SLDATKIFAQAIENGDADQLDDSSSESDTDMTKELDSNDDNSLDNSELQISPIKNGIMKK  780
              ||||||||||||||||||||||||||||||||||||||||||||||||||||||||||||
Dnmt3ba       SLDATKIFAQAIENGDADQLDDSSSESDTDMTKELDSNDDNSLDNSELQISPIKNGIMKK  780

Dnmt3ba       SRRSTSETVQHSNAVKPCVVRLEKITSQREVNLKNDGAKNDGTLTSNTLKEVKNDLSNST  840
              ||||||||||||||||||||||||||||||||||||||||||||||||||||||||||||
Dnmt3ba       SRRSTSETVQHSNAVKPCVVRLEKITSQREVNLKNDGAKNDGTLTSNTLKEVKNDLSNST  840
```

```
Dnmt3ba      PSPRRGKRDQAYNDGKGFAVGELVWGKVKDFSLWPGLVVPWKGRIVPVSMRRVEWFGDGM  900
             ||||||||||||||||||||||||||||||||||||||||||||||||||||||||||||
Dnmt3ba      PSPRRGKRDQAYNDGKGFAVGELVWGKVKDFSLWPGLVVPWKGRIVPVSMRRVEWFGDGM  900

Dnmt3ba      FSEIHTDGLLPFGAFSKNFCSKSYEGLPTYKNAIYQILELAAERSGKLFPPSEKKGEEVK  960
             ||||||||||||||||||||||||||||||||||||||||||||||||||||||||||||
Dnmt3ba      FSEIHTDGLLPFGAFSKNFCSKSYEGLPTYKNAIYQILELAAERSGKLFPPSEKKGEEVK  960

Dnmt3ba      AMMDWAFGGFQPMGADGFLPSADSSASNKTESDSSVSDYQPPAKRKYVFKNRPSTQECNR  1020
             ||||||||||||||||||||||||||||||||||||||||||||||||||||||||||||
Dnmt3ba      AMMDWAFGGFQPMGADGFLPSADSSASNKTESDSSVSDYQPPAKRKYVFKNRPSTQECNR  1020

Dnmt3ba      DQMVQEVTSKGRKIEDFCLSCGSSNTEIFHPLFKGSLCIKCKENFTETLYRYDDDGYQSY  1080
             ||||||||||||||||||||||||||||||||||||||||||||||||||||||||||||
Dnmt3ba      DQMVQEVTSKGRKIEDFCLSCGSSNTEIFHPLFKGSLCIKCKENFTETLYRYDDDGYQSY  1080

Dnmt3ba      CTVCCAGLEVILCGNASCCRCFCKDCLNVLVGPGTFDKLKEVDPWSCYVCLPSKCYGVLK  1140
             ||||||||||||||||||||||||||||||||||||||||||||||||||||||||||||
Dnmt3ba      CTVCCAGLEVILCGNASCCRCFCKDCLNVLVGPGTFDKLKEVDPWSCYVCLPSKCYGVLK  1140

Dnmt3ba      LRTDWSVRVQEFFANNSAFEFEPHRVYPSIPAHKRRPIRVLSLFDGIATGYLVLKDLGFK  1200
             ||||||||||||||||||||||||||||||||||||||||||||||||||||||||||||
Dnmt3ba      LRTDWSVRVQEFFANNSAFEFEPHRVYPSIPAHKRRPIRVLSLFDGIATGYLVLKDLGFK  1200

Dnmt3ba      LERYIASEICEDSIAVGMVKHEGKIEYVKDVRTITRKHLAEWGPFDLLIGGSPCNDLSMV  1260
             |||||||||||||||||||||||||||||||||||||||||||||||||||||||
Dnmt3ba      LERYIASEICEDSIAVGMVKHEGKIEYVKDVRTITRKHLAEWGPFDLLIGGSPCNVMVHG  1260

Dnmt3ba      NPARKGLFEGTGRLFFEYYRMLTMMRPKEDDDRPFFWLFENVVAMSAHDKADICRFLECN

Dnmt3ba      ESSQKRSF* 1268

             EGTGRLFFEYYRMLTMMRPKEDDDRPFFWLFENVVAMSAHDKADICRFLECNPVMIDAVK

             VSPAVSPAHRARYFWGNLPGMNRPVATSLTDNVDLQDCLESGRTAMFSKVRTITTKSNSI

             KQGKTGPLPVTMNGKEDYLWCTEMEKIFGFPKHYTDVNNMGRGQRQKVLGRSWSVPVIRH

             LFAPLKDYFACE*
```
